# MiR-25 Protects Cardiomyocytes against Oxidative Damage by Targeting the Mitochondrial Calcium Uniporter

**DOI:** 10.3390/ijms16035420

**Published:** 2015-03-10

**Authors:** Lei Pan, Bi-Jun Huang, Xiu-E Ma, Shi-Yi Wang, Jing Feng, Fei Lv, Yuan Liu, Yi Liu, Chang-Ming Li, Dan-Dan Liang, Jun Li, Liang Xu, Yi-Han Chen

**Affiliations:** 1 Key Laboratory of Arrhythmias of the Ministry of Education of China, East Hospital, Tongji University School of Medicine, Shanghai 200120, China; E-Mails: panlei_east@126.com (L.P.); huangbijun56@126.com (B.-J.H.); maxiue1989@126.com (X.-E.M.); wangshiyi840101@sina.com (S.-Y.W.); afenger@126.com (J.F.); lvfeihom@163.com (F.L.); liuyuan_wy@163.com (Y.L.); yiliu@tongji.edu.cn (Y.L.); lichangming@outlook.com (C.-M.L.); dandanliang_tj@hotmail.com (D.-D.L.); junli@tongji.edu.cn (J.L.); 2 Research Center for Translational Medicine, East Hospital, Tongji University School of Medicine, Shanghai 200120, China; 3 Institute of Medical Genetics, Tongji University, Shanghai 200092, China; 4 Department of Cardiology, East Hospital, Tongji University School of Medicine, Shanghai 200120, China; 5 Department of Pathology and Pathophysiology, Tongji University School of Medicine, Shanghai 200092, China

**Keywords:** cardiomyocytes, miR-25, mitochondrial calcium uniporter, oxidative stress

## Abstract

MicroRNAs (miRNAs) are a class of small non-coding RNAs, whose expression levels vary in different cell types and tissues. Emerging evidence indicates that tissue-specific and -enriched miRNAs are closely associated with cellular development and stress responses in their tissues. MiR-25 has been documented to be abundant in cardiomyocytes, but its function in the heart remains unknown. Here, we report that miR-25 can protect cardiomyocytes against oxidative damage by down-regulating mitochondrial calcium uniporter (MCU). MiR-25 was markedly elevated in response to oxidative stimulation in cardiomyocytes. Further overexpression of miR-25 protected cardiomyocytes against oxidative damage by inactivating the mitochondrial apoptosis pathway. MCU was identified as a potential target of miR-25 by bioinformatical analysis. MCU mRNA level was reversely correlated with miR-25 under the exposure of H_2_O_2_, and MCU protein level was largely decreased by miR-25 overexpression. The luciferase reporter assay confirmed that miR-25 bound directly to the 3' untranslated region (UTR) of MCU mRNA. MiR-25 significantly decreased H_2_O_2_-induced elevation of mitochondrial Ca^2+^ concentration, which is likely to be the result of decreased activity of MCU. We conclude that miR-25 targets MCU to protect cardiomyocytes against oxidative damages. This finding provides novel insights into the involvement of miRNAs in oxidative stress in cardiomyocytes.

## 1. Introduction

Myocardial cell death often occurs under conditions of oxidative stress, such as ischemia reperfusion and myocardial infarction [1,2]. Hallmark events including mitochondrial permeability transition pore (mPTP) opening and dissipation of the mitochondrial inner membrane potential are usually initiated by excessive mitochondrial Ca^2+^ overload [3]. One important approach to cardioprotection against oxidative stress injury is to reduce oxygen consumption and inhibit apoptosis in cardiomyocytes [4].

MicroRNAs (miRNAs) are small non-coding RNAs that inhibit the translation of target mRNAs and/or cause their destabilization through imperfect sequence-specific binding to the 3'-untranslated region (UTR) of target mRNAs. Increasing evidence shows that miRNA expression is tightly regulated in a tissue-specific and a time-dependent manner [5,6]. For example, miR-l, miR-133, miR-21, miR-206 and miR-199 are highly and specifically expressed in cardiovascular tissues [7,8,9]. These cardiac-specific miRNAs are involved in the regulation of multiple physiological and pathological processes, such as cardiac morphogenesis, heart failure, and arrhythmias [10,11].

In the present study, we demonstrate that miR-25, a cardiac-enriched miRNA, is sensitive to oxidative stress. The overexpression of miR-25 can protect cardiac myocytes against oxidative injury. Mechanistically, miR-25 directly down-regulates the expression of mitochondrial MCU and inhibits the increase of the mitochondrial Ca^2+^ and mitochondrial apoptosis pathway.

## 2. Results

### 2.1. MiR-25 Was Remarkably Elevated under Oxidative Stress

**Figure 1:**
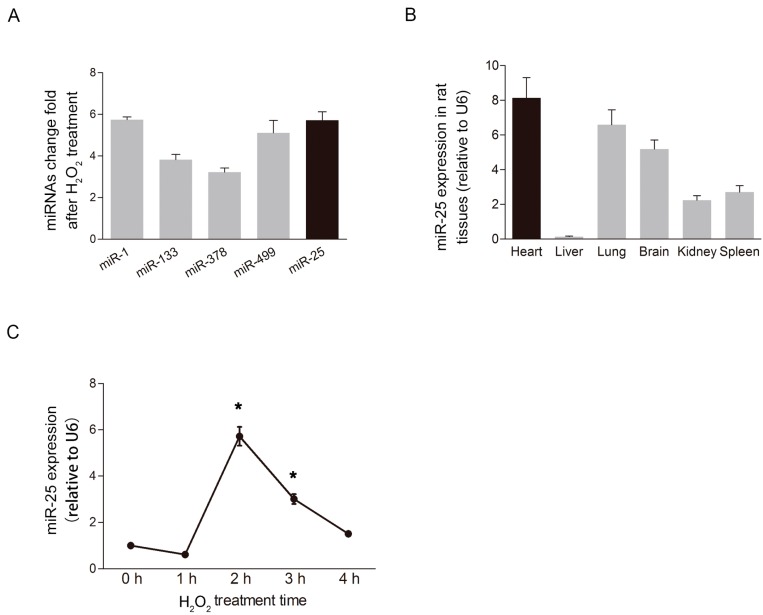
MiR-25 was elevated under oxidative stress. (**A**) MiR-25 expression was dramatically altered in response to oxidative stress compared to other miRNAs that are highly expressed in cardiac tissue (*n* = 3); (**B**) Quantitative RT-PCR confirmed the expression profile of miR-25 in different rat organs (*n* = 3); (**C**) Time course of the relative expression of miR-25 in cardiomyocytes in response to H_2_O_2_ stimulation (500 μM). The expression of miR-25 increased significantly at 2 h, and gradually returned to baseline by 4 h. Data are means ± SD from three independent experiments. * *p* < 0.05 *vs.* control (0 h).

To screen out the cardiac-enriched miRNAs involved in oxidative stress, we used H9c2 cells, an embryonic rat ventricular myocyte cell line, and examined miRNA expression levels in cells exposed to oxidative stress using microarray analysis (GSE36239 and GSE55791) [12,13]. The oxidative stress was induced by 4 h of incubation with H_2_O_2_, an exogenous source of reactive oxygen species (ROS) that increases lipid peroxidation, and decreases antioxidant activity and damages cardiomyocytes [14,15,16]. The results indicated that the most significant change in response to oxidative stress was observed for miR-25 (Figure 1A). Next, we used RT-PCR to confirm the expression profile of miR-25 in different rat organs (Figure 1B). The highest expression level of miR-25 was in the heart. Finally, to verify the reaction to H_2_O_2_ stimulation, we monitored the expression of miR-25 at five different time points, and we found that the expression of miR-25 exposed to H_2_O_2_ in cardiomyoctye was time-dependent (Figure 1C). Following oxidant stimulation, the levels of miR-25 in H9c2 cells remained unchanged within 1 h, dramatically increased more than five-fold at 2 h, and then gradually returned to control levels.

### 2.2. Over-Expression of MiR-25 Protected Cardiomyocytes against Oxidative Damage by Inactivating the Mitochondrial Apoptosis Pathway

**Figure 2:**
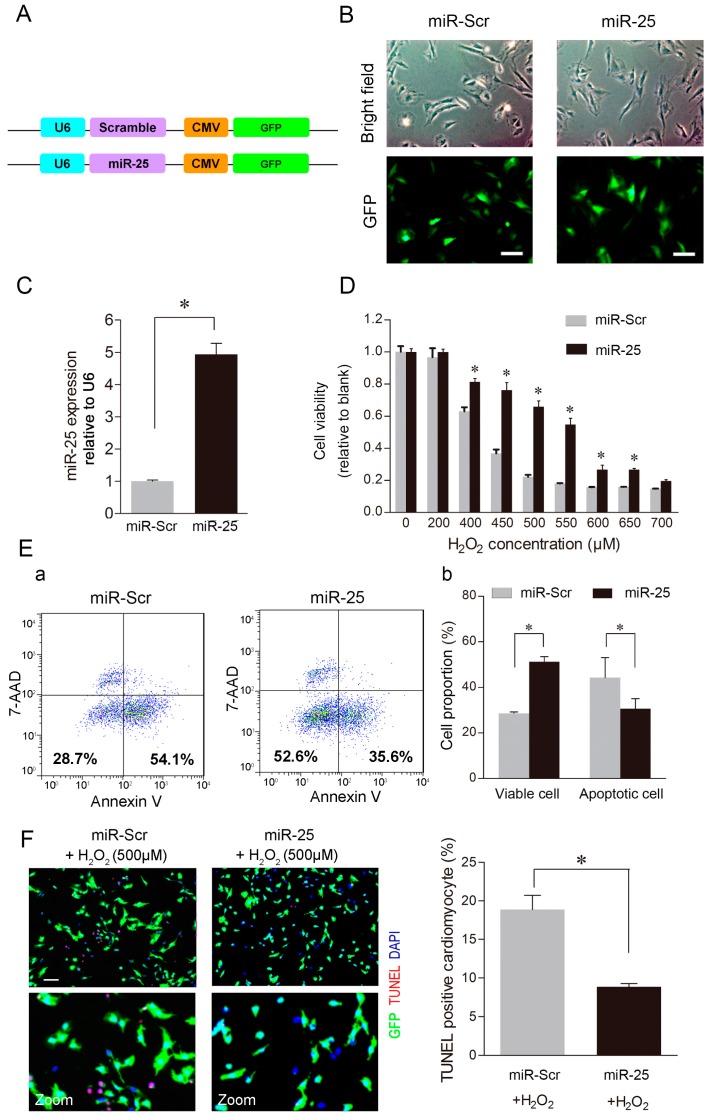
Over-expression of miR-25 markedly reduced cardiomyocyte apoptosis. (**A**) Schematic illustration of the construction of the lentivirus vectors. H9c2 cells infected with lentiviruses containing miR-25 precursor (miR-25) or a scramble control sequence (miR-Scr) were isolated by fluorescence-activated cell sorting (FACS) based on the green fluorescence emitted from cells expressing GFP, the marker carried by the lentivirus vector, and then plated in 24-well plates for expansion; (**B**) The phase contrast and fluorescent images of H9c2 cells infected with lentiviruses containing miR-25 or miR-Scr. Scale bars: 100 μm; (**C**) Quantitative RT-PCR showed that miR-25 expression was 4.94 ± 0.34-fold higher in the miR-25 group than that in the miR-Scr group. * *p* < 0.05 *vs.* miR-Scr; (**D**) H9c2 cells were stimulated with H_2_O_2_ and subjected to CCK-8 assay. Serial concentrations of H_2_O_2_ were applied to examine the effects of miR-25 overexpression; (**E**) Panel **a**: representative dot-plot diagrams of FACS analysis on H9c2 cells stained with annexin V and 7-AAD. The lower **left** quadrants represents the viable cells (Annexin V^−^/7-AAD^−^), and the lower **right** quadrants represents the apoptotic cells (Annexin V^+^/7-AAD^−^); Panel **b**: percentages of viable and apoptotic cells; (**F**) Representative images and quantification of the TUNEL assay. Apoptotic cells were stained red by the TUNEL assay. Scale bars: 100 μm. Data are means ± SD from three independent experiments. * *p* < 0.05 *vs.* respective control.

To evaluate the physiological function of miR-25, H9c2 cells were infected with a lentivirus carrying the miR-25 precursor (miR-25) or a scramble sequence (miR-Scr) (Figure 2A). The infected cardiomyocytes express green fluorescence protein (GFP), which can be observed by microscopy, as well as sorted by FACS. Under the light and fluorescence microscopy, no morphological difference was observed between cells expressing miR-25 and miR-Scr (Figure 2B). Real-time PCR analysis showed that the expression level of miR-25 was 4.94 ± 0.34-fold higher in the miR-25 group than that in the miR-Scr group (Figure 2C).

Next, both miR-25 and miR-Scr cells were treated with H_2_O_2_, and then cell vitality assay was performed. The results showed that miR-25 protected more H9c2 cells at different H_2_O_2_ concentrations, compared to the miR-Scr control (Figure 2D). This pro-survival effect was especially prominent at concentrations of H_2_O_2_ ranging from 450–550 μM, in which miR-25 enhanced cell viability by two to three-fold as shown by the CCK-8 assay. In addition, FACS analysis using annexin V and 7-AAD staining showed that there were fewer apoptotic cells (annexin V^+^/7-AAD^−^, 30.6% ± 3.31% *vs.* 44.3% ± 6.53%; *p* < 0.05) and more live cells (annexin V^−^/7-AAD^−^, 51.3% ± 1.71% *vs.* 28.6% ± 0.42%; *p* < 0.05) in the miR-25 group than those in the miR-Scr group after 4-h exposure of H_2_O_2_ (500 μM) (Figure 2E). In line with the results of CCK-8 assay and FACS analysis, the *in situ* TUNEL assay showed that the number of apoptotic cells in the miR-Scr group was double of that in the miR-25 group after the treatment with H_2_O_2_ (Figure 2F). These data suggested that miR-25 reduced cardiomyocyte apoptosis.

**Figure 3:**
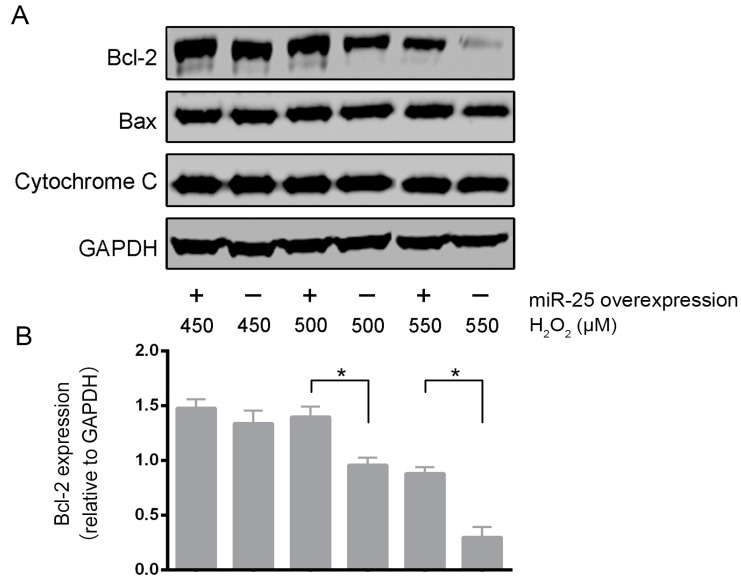
MiR-25 protected cardiomyocytes by inhibiting the mitochondrial apoptosis pathway. (**A**) Representative Western blot images showing the expression levels of Bcl-2, Bax and cytochrome C in H9c2 cells infected with miR-25 lentiviruses; (**B**) The quantitative data of the Western blot. GAPDH served as a loading control. Data are means ± SD from three independent experiments. * *p* < 0.05.

We further examined the expression of key proteins involved in the mitochondria-dependent apoptosis pathway (Figure 3). With the increment of H_2_O_2_ concentrations, the anti-apoptotic protein Bcl-2 was decreased, whereas the pro-apoptotic proteins Bax and cytochrome C remained unchanged. Compared to the control group, miR-25 overexpression preserved Bcl-2 protein levels in cells exposed to high concentrations of H_2_O_2_ (500–550 μM), but had no effects on the levels of Bax and cytochrome C. Bcl-2 is a key anti-apoptotic molecule. These results support that miR-25 protects cardiomyocytes by inhibiting apoptosis.

### 2.3. MCU Was a Direct Target of MiR-25

**Figure 4:**
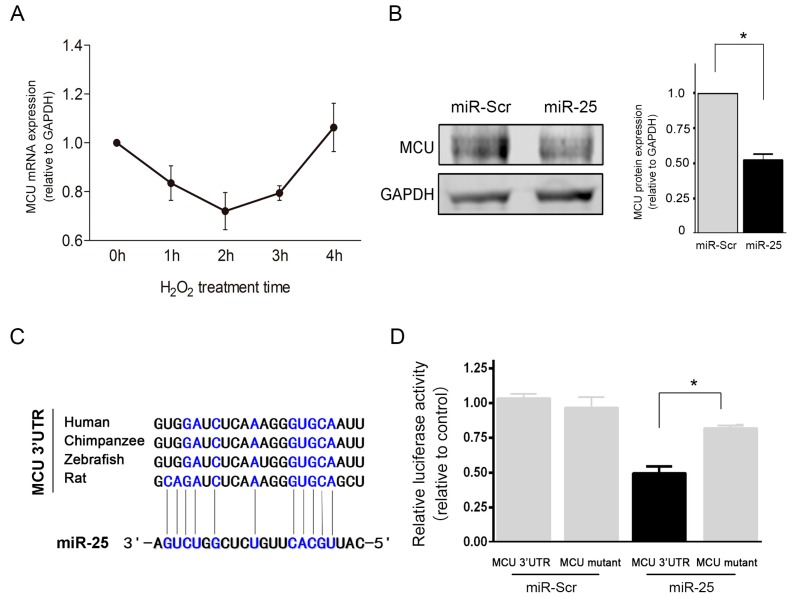
MCU is directly targeted by miR-25. (**A**) The time-course changes in the expression of MCU mRNA in response to H_2_O_2_ exposure; (**B**) Western blot analysis of MCU in H9c2 cells overexpressing miR-25 or scramble control sequence (miR-Scr); (**C**) The potential binding site between miR-25 and the 3'-UTR of MCU mRNA. The complementary nucleotides are indicated by bolded blue letters; (**D**) The luciferase assay was performed using reporter plasmids harboring the wild-type and mutant MCU 3'-UTR. MiR-25 significantly inhibited the luciferase activity of the wild-type reporter, but not that of the mutant reporter. * *p* < 0.05.

To identify the mechanism underlying miR-25-mediated anti-apoptosis, potential target genes of miR-25 were predicted using bioinformatics algorithms. Mitochondrial fission factor (MFF), mitochondrial calcium uniporter (MCU), and NADPH oxidase 4 (Nox4) were identified as putative targets of miR-25. To verify their validity, the relative expression levels of these targets were detected after H_2_O_2_ exposure. Only the mRNA level of MCU showed a significant negative correlation with miR-25 (Figure 4A). The overexpression of miR-25 also significantly reduced the protein level of MCU, which likely accounted at least in part, for its anti-apoptotic function (Figure 4B). Additionally, miR-25 significantly reduced the luciferase activity of wild-type reporters harboring MCU 3'-UTR by about 50% (*p* < 0.05), compared to the miR-Scr control (Figure 4C,D). In contrast, the mutant luciferase reporter with three nucleotide mutations in the potential binding area of MCU 3'-UTR to miR-25 seed sequence was rescued, demonstrating that the seed sequence of miR-25 directly mediated the repression of MCU expression.

### 2.4. MiR-25 Significantly Decreased H_2_O_2_-Induced Elevation of Mitochondrial Ca^2+^ Concentration

**Figure 5:**
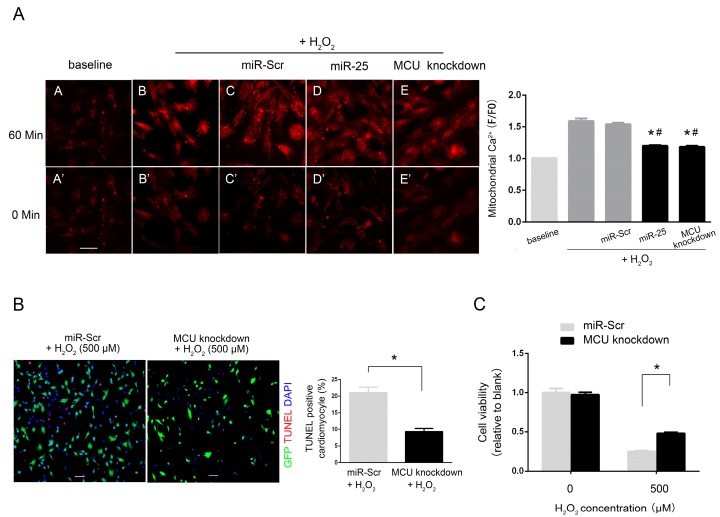
MiR-25 over-expression and MCU knockdown individually blocked H_2_O_2_-induced mitochondrial Ca^2+^ elevation in H9c2 cells. Mitochondrial Ca^2+^ was determined by Rhod-2 AM, a mitochondrial-specific Ca^2+^ probe, after the treatment of 500 μM H_2_O_2_ for 60 min. Cells were infected with a lentivirus carrying the miR-25 precursor (miR-25) or a scramble control sequence (miR-Scr) or MCU shRNA (knockdown). (**A**) Representative images (**left**) and quantitative analysis (**right**) of cells stained with Rhod-2 AM. Scale bar: 50 μm; (**B**) Representative images (**left**) and quantitative analysis (**right**) of the TUNEL assay. Apoptotic cells were stained red by the TUNEL assay. Scale bar: 100 μm; (**C**) Cell viability of H9c2 cells stimulated with H_2_O_2_, determined by CCK-8 assay. Data are means ± SD from three independent experiments. * *p* < 0.05 *vs.* H_2_O_2_ + miR-Scr, ^#^ *p* < 0.05 *vs.* H_2_O_2_.

MCU is a critical mitochondrial Ca^2+^ transporter that regulates mitochondrial Ca^2+^ concentration. Based on the above observation that MCU is a target of miR-25, we examined whether miR-25 regulates mitochondrial Ca^2+^ homeostasis, using a calcium imaging technique. As shown in Figure 5, mitochondrial Ca^2+^ in H9c2 cells was determined by Rhod-2 AM at 60 min after the treatment of H_2_O_2_ (500 μM). Increases in mitochondrial Ca^2+^ were observed in cells exposed to H_2_O_2_. The over-expression of miR-25 significantly attenuated such increases, demonstrating the inhibition of mitochondrial Ca^2+^ by miR-25 (Figure 5A). In addition, the knockdown of MCU inhibited H_2_O_2_-induced rises in mitochondrial Ca^2+^, which is in accordance with the basic function of MCU (Figure 5A). MCU knockdown also reduced cell apoptosis (Figure 5B) and enhanced cell viability (Figure 5C). Taken together, the inhibition of miR-25 on mitochondrial Ca^2+^ elevation under oxidative stress supports the role of MCU as a target for miR-25.

## 3. Discussion

The present study provides evidence for the anti-apoptotic effects of miR-25 by decreasing MCU expression, relieving mitochondrial Ca^2+^ overload and inactivating the mitochondrial apoptosis pathway in cardiomyocytes exposed to oxidative stress. First, among the cardiac-enriched miRNAs, miR-25 was identified as a sensitive responder to oxidative stress. Second, overexpression of miR-25 conferred a significant resistance to oxidative damage by inhibiting the mitochondrial apoptosis pathway. Third, among the potential target genes of miR-25, the expression of MCU in cardiomyocytes was reversely correlated with that of miR-25 after H_2_O_2_ exposure, and MCU was verified by the luciferase reporter assay to be a direct target of miR-25. Fourth, we observed that miR-25 alleviated mitochondrial Ca^2+^ elevation induced by H_2_O_2_, while MCU is a well-known regulator of mitochondrial Ca^2+^ homeostasis. These findings suggest that miR-25 in cardiomyocytes plays a protective role in oxidative stress by regulating Ca^2+^ homeostasis and the apoptosis pathway.

MiRNAs are emerging as critical regulators in physiological and pathogenetic processes. Physical and chemical stimuli may change miRNA expression [17,18]. The present study examined the expression levels of cardiac-enriched miRNAs in response to oxidative stimulation. Our results showed that miR-1 and miR-25 changed most dramatically. Since multiple studies have addressed the role of miR-1 in the heart [19,20], the present study made further observations on miR-25, but not miR-1. To uncover the role of miR-25 in oxidative stress, miR-25 was overexpressed using lentiviruses to observe its effect on the damages caused by the oxidative stress inducer H_2_O_2_. To address this question, we used a series of concentration of H_2_O_2_, ranging from a low dose (200 nM) to a high dose (700 nM), which undoubtedly induced necrosis and apoptosis in H9c2 cells. The most suitable and efficient concentration of H_2_O_2_ for inducing oxidative stress was established to be 500 nM in our settings, in accordance with the concentration documented to induce apoptosis in H9c2 cells [21]. By determining cell viability with CCK-8 assay and apoptotic cells with FACS and TUNEL analyses, we observed the anti-apoptotic effect of miR-25, which was most significant under the stimulation of 500 nM H_2_O_2_. The major mechanism through which miRNAs inhibit target gene expression is to induce mRNA degradation and/or mRNA translation suppression via the imperfect matching to the 3'-UTR of target mRNAs [17]. Here, we used bioinformatics analysis to search the potential targets of miR-25, with the focus on target genes involved in mitochondrial apoptosis pathway. MCU was selected as a potential target for further mechanical study. We found that MCU mRNA levels were negatively correlated with those of miR-25. Consistently, MCU protein levels were greatly reduced by miR-25 overexpression. The luciferase assay further confirmed that MCU is a direct target of miR-25. MCU is a 40-kDa protein that is sensitive to ruthenium-red-sensitive mitochondrial Ca^2+^ uptake [22]. It is a pivotal member of mitochondrial calcium uniporter complex, comprising of mitochondrial Ca^2+^ uptake1 (MICU1), MICU2, MCU and essential MCU regulator (EMRE) [23]. The MCU complex has been demonstrated to control the Ca^2+^ flux across the inner mitochondrial membrane [24,25]. Ca^2+^ homeostasis is essential for the maintenance of the physiological activity of the heart. It regulates energy production, modulates cytoplasmic Ca^2+^ signaling, and switches the transition from reversible to irreversible cell death [26,27]. There is evidence that an increased MCU current results in mPTP opening and myocardial cell death. Specific inhibition of MCU with Ru360 provided cardioprotective effects, while activation of MCU with spermine abolished this beneficial activity [28,29]. MCU is a target that promotes cardiomyocyte survival during mitochondrial stress [30]. The present study identified MCU as a direct target gene of miR-25. Based on the aforementioned function of MCU, it is presumable that miR-25 overexpression may facilitate mitochondrial Ca^2+^ homeostasis. By using a mitochondria-specific Ca^2+^ dye, we did observe that the increase of mitochondrial Ca^2+^ induced by oxidative stimulation was attenuated by miR-25 over-expression. MCU knockdown also relieved mitochondrial Ca^2+^ overload and reduced cell apoptosis under oxidative stress. These results are consistent with previous reports that miR-25 can inhibit mitochondrial Ca^2+^ uptake in Hela cells [31], and that knockdown of MCU markedly reduced mitochondrial Ca^2+^ uptake [22]. Taken together, it is suggested that miR-25 regulates mitochondrial Ca^2+^ homeostasis by targeting MCU in cardiomyocytes. This is the first time that miR-25-mediated MCU downregulation is observed to be correlated with the anti-apoptotic activity in cardiomyocytes.

SERCA was recently shown to be down-regulated by miR-25 in the failing heart, but other calcium handling proteins, including the sodium-calcium exchanger (NCX1), calmodulin kinase-2 (CaMKII), phospholamban (PLN) and Calmodulin-3 (Calm3) were unaffected. Downregulation of SERCA leads to the dysfunction of Ca^2+^ re-uptake and the elevation of Ca^2+^ in the cytoplasm, which is harmful to cardiomyocytes [32]. Meanwhile, the elevated cytosolic Ca^2+^ may enter mitochondria and cause mitochondrial Ca^2+^ overload and subsequent cell dysfunction. Our present finding that the overexpression of miR-25 conferred a significant resistance to oxidative damage in cardiomyocytes appears to be different from the above scenario. A potential reason might be that miR-25-mediated regulation of MCU protected the mitochondria from oxidative stress, which well counteracted the detrimental cytosolic Ca^2+^ overload by the reduced SERCA protein. Bcl-2 protein is acknowledged as one of the key regulators of programmed cell death [33]. The present study observed that miR-25 over-expression rescued Bcl-2 expression in oxidative stress. However, there is no evidence that Bcl-2 is directly regulated by MCU. We think that the increase of Bcl-2 expression might be subsequent to the inhibition of MCU by miR-25. Buffering mitochondrial Ca^2+^ was found to increase the expression of Bcl-2 in hepatocytes [34]. Therefore, it is possible that miR-25 facilitates Bcl-2 expression by attenuating mitochondrial Ca^2+^ overload in oxidative stress. Further study needs to be done to address this issue.

## 4. Methods and Materials

### 4.1. Cell Culture and Viral Infection

The rat cardiac myoblast H9c2 cell line was cultured in DMEM supplemented with 10% fetal bovine serum (Hyclone Laboratories, Logan, UT, USA), 2 mM l-glutamine, and penicillin/streptomycin (Invitrogen, Carlsbad, CA, USA) in a humidified 5% CO_2_ incubator at 37 °C. The plv-miR vector was provided by Jian-hua Wang (Shanghai Jiaotong University School of Medicine, Shanghai, China). The construction of lentiviral vectors containing miR-25 precursor, MCU interference sequence (GCCAGAGACAGACAATACT) and scramble sequence (TTCTCCGAACGTGTCACGT) were generated by insertion of corresponding sequences. Replication-incompetent lentiviral particles were packaged in 293T cells with helper plasmids (gag-pol and VSV-G) and used to infect H9c2 cells.

### 4.2. Quantitative Reverse Transcription-Polymerase Chain Reaction

Total RNA was isolated using the mirVana kit (for miRNA; Ambion, Austin, TX, USA) or with TRIzol (mRNA; Invitrogen, Carlsbad, CA, USA). MiRNA was quantified by reverse transcription-polymerase chain reaction (qRT-PCR; Applied Biosystems, Foster City, CA, USA). Cardiac-enriched miRNAs with RNU-6B were quantified by SYBR Green detection and miScript primer assays (Qiagen, Valencia, CA, USA). The mRNA quantification was conducted using SYBR Green-based detection technology. The primers were as follows: rat MCU: forward 5'-AGATGGTGTTCGAGTTGCTG-3'; reverse 5'-AGGGTCTCTGCGTTTTCATG-3'. GAPDH: forward 5'-CTGGTGCTGAGTATGTCGTGGA-3'; reverse 5'-AGTTGGTGGTGCAG-GATGCATT-3'. The expression of each miRNA under the baseline and the exposure of H_2_O_2_ was first normalized to the internal reference (∆*C*_t_) and then, the fold change of miRNAs in H_2_O_2_ group was compared to that of the baseline (∆∆*C*_t_) and expressed as 2^−(∆∆*C*t)^.

### 4.3. Fluorescence-Activated Cell Sorting (FACS)

The apoptosis was determined by FACS assay. The H9c2 cells infected with recombinant lentivirus were digested with 0.25% trypsin-EDTA solution (Invitrogen, Carlsbad, CA, USA) for 5 min at 37 °C and then resuspended in the culture medium to obtain a single cell suspension. The detector settings were calibrated using uninfected H9c2 cells that were likewise digested. The sorted cells were plated in gelatin-coated 24-well plates at a density of 10^5^ cells/well. For the apoptosis analysis, H_2_O_2_ (Sigma, St. Louis, MO, USA)-treated H9c2 cells were washed with calcium buffer and incubated with annexin V and 7-AAD for 10 min and then analyzed using a BD FACSAria II (BD Biosciences, San Jose, CA, USA).

### 4.4. Cell Vitality Analysis

Cell vitality was determined using CCK-8 dye (Beyotime, Zhejiang, China) according to the manufacturer’s instructions. Briefly, 5 × 10^3^ cells/well were seeded in a 96-well flat-bottomed plate and then cultured at 37 °C for 24 h. Subsequently, the cells were treated with H_2_O_2_ at increasing concentrations (200, 400, 450, 500, 550, 600, 650, 700 μM) in the presence of 10% FBS for 4 h. After the addition of 10 μL CCK-8 dye to each well, the cells were incubated at 37 °C for further 2 h and then the absorbance was determined at 450 nm using a microplate reader.

### 4.5. TUNEL Assay

Apoptosis was induced with H_2_O_2_ from Sigma (Sigma, St. Louis, MO, USA). After exposure to 500 μM H_2_O_2_ for 4 h, cells cultured on coverslips (1 × 10^5^ cells/well of a 24-well plate) were used for apoptotic TUNEL detection using the *in situ* Cell Death Detection Kit (Roche Diagnostics, Indianapolis, IN, USA) and analyzed by fluorescence microscopy.

### 4.6. Immunoblotting

Protein lysates for immunoblotting were prepared in RIPA buffer (Beyotime, Zhejiang, China) containing protease inhibitor cocktail tablets (Roche Applied Science, Mannheim, Germany), and the protein concentrations were quantified using the BCA protein assay kit (Pierce, Rockford, IL, USA). Western blot analysis was performed using standard techniques. The primary antibodies used were MCU (1:200; Santa Cruz, CA, USA), Bcl-2, Bax, Cytochrome (1:1000; Cell Signaling, Danvers, MA, USA) and GAPDH (1:5000; Beyotime, Zhejiang, China). The appropriate IRDye 800-conjugated secondary antibodies were used (1:10,000; LI-COR Biosciences, Lincoln, NE, USA) to visualize the blotting. Images were recorded using the Odyssey infrared imaging system and analyzed with Odyssey Application Software v2 (LI-COR Biosciences).

### 4.7. Luciferase Reporter Assay

TargetScan and Pictar were used to predict the miR-25 binding sites within MCU. The MCU 3'-UTR was amplified from rat genomic DNA using the following primers: forward 5'-CCGTTGTTTAAACTCAGAGATGAGCGTGAATCCCG-3'; reverse 5'-CGGAAGCGGCCGCCCCTGCAAAGATGCACGAAGC-3'.

The 301-base-pair product was cloned and inserted into the PmeI/NotI sites of psiCheck2 (Promega, Madison, WI, USA). The resultant p3'UTR-MCU plasmids were mutated by PCR using primers designed to incorporate a three-base substitution into the miR-25 seed sequence (5'-GAATCCAA***GTG***CAA converted to 5'-GAATCCAA***CAC***CAA). The resulting pMut-3'UTR-MCU plasmids were co-transfected with miR-25. Forty-eight hours later, the firefly and Renilla luciferase activities were analyzed using the Dual-Luciferase Reporter Assay System (Promega, Madison, WI, USA).

### 4.8. Mitochondrial Calcium Recordings and Analyses

Mitochondrial calcium concentrations in H9c2 cells were measured by the mitochondria-selective Ca^2+^ indicator Rhod-2 AM. H9c2 cells over-expressed with miR-25, MCU-shRNA or scramble miRNA were grown on glass slides and loaded with 5 μM Rhod-2 AM, followed by H_2_O_2_ treatment (500 μM) for 60 min at room temperature. The fluorescence intensities were immediately analyzed with a Nikon inverted fluorescence microscope. Ca^2+^ transients are presented as the fluorescence ratio, normalized to the background intensity (F/F0).

### 4.9. Statistical Analysis

All of the data are presented as the means ± SD. Differences between multiple groups were analyzed with one-way ANOVA followed by the Fisher’s least significant difference (LSD) test. Differences between two groups were analyzed with Student’s *t*-test. A *p* < 0.05 was considered statistically significant.

## 5. Conclusions

In summary, our observations provide evidence that miR-25 protects cardiomyocytes against oxidative damage by reducing the expression of MCU, and subsequently attenuating mitochondrial Ca^2+^ overload and apoptosis. We first addressed the role of miR-25 in the cardiomyocytes. Our findings provide novel insights for miRNA in defending against oxidative stress, which might represent a novel target for the treatment of heart diseases associated with oxidative stress.
